# A Double-Site Chemodosimeter for Selective Fluorescence Detection of a Nerve Agent Mimic

**DOI:** 10.3390/molecules27020489

**Published:** 2022-01-13

**Authors:** Xin Guo, Chang-Xiang Liu, Yuan Lu, Ya-Wen Wang, Yu Peng

**Affiliations:** 1 School of Life Science and Engineering, Southwest Jiaotong University, Chengdu 610031, China; guoxin@my.swjtu.edu.cn (X.G.); lchx2020211799@my.swjtu.edu.cn (C.-X.L.); 2 Chengdu Municipal Bureau of Economic and Information Technology, Chengdu 610229, China; luyuan@lzu.edu.cn

**Keywords:** fluorescence, diethyl chlorophosphate (DCP), double-site, chemodosimeter

## Abstract

A novel two-site chemodosimeter (**SWJT-4**) based on fluorescein skeleton to detect diethyl chlorophosphate (DCP) was designed and synthesized. It is a turn-on fluorescent probe for DCP with good selectivity and obvious color change in aqueous solution. Interestingly, the two oxime groups of **SWJT-4** as dual response sites initiated different reactions with DCP to form a cyano group and an isoxazole ring, respectively. The corresponding mechanism was confirmed by ^1^H NMR, MS and DFT calculation. Moreover, **SWJT-4** could be used as a fluorescent test paper to detect DCP vapor.

## 1. Introduction

Organophosphate nerve agents refer to a class of chemical substances composed of organophosphorus compounds (OPs). They have been widely used in pesticides and chemical warfare agents, such as parathion, systemic phosphorus, malathion, dimethoate, DDVP, tabun, sarin and soman [1,2]. These chemicals can result in a range of neurological symptoms such as headache, dizziness and agitation [3,4,5,6]. Organophosphorus compounds were used as a chemical weapon in war to threaten the safety of human life seriously. In a word, organophosphorus nerve agents are not only a potential threat of biochemical warfare, but also a usual weapon of terrorist organizations. Therefore, the detection of these substances by a convenient method is very necessary.

At present, the fluorescence analysis methods have attracted widespread attention due to their real-time monitoring, high selectivity, high sensitivity, low detection limit and so on. To obtain specific recognition of diethyl chlorophosphate (DCP), an organophosphate nerve agent mimic, some fluorescence probes were designed as chemodosimeters based on organic reaction [7,8,9,10,11,12,13,14,15,16,17,18,19,20,21,22,23,24,25,26,27,28,29,30]. Notably, DCP can phosphorylate with such probes including amino [7,8,9], hydroxyl [10,11,12,13,14,15,16], oxime [17,18,19,20,21] and pyridine [22,23,24] groups as the reaction sites. DCP can be reacted with a hydroxyl-ammonia [25,26] or carboxyl-ammonia [27] group of probes to produce cyclization products as well. The different structures of the probe and the corresponding product lead to the changes in fluorescent signals. However, most probes for DCP are fluorescence ON-OFF responses [31,32,33,34,35,36,37]. In addition, several probes detect DCP in organic solution [17,20,25,28], but a few probes detect DCP in aqueous solution (Table S1, Supplementary Materials) [18,38,39]. Therefore, the development of a turn-on fluorescent probe for DCP in an aqueous solution is still in high demand.

In our previous work, we synthesized a series of DCP probes based on ON-OFF fluorescent responses [40,41]. In the present work, a novel fluorescent probe **SWJT-4** with two response sites was designed and synthesized for selective detection of DCP. It has good photochemical stability in an aqueous solution. The turn-on fluorescent responses could be achieved by tethering two oxime groups to a fluorescein skeleton. Moreover, **SWJT-4** could be used to detect DCP vapor by a fluorescent test paper.

## 2. Results and Discussion

### 2.1. Design and Photoproperties of **SWJT-4**

**Scheme 1:**
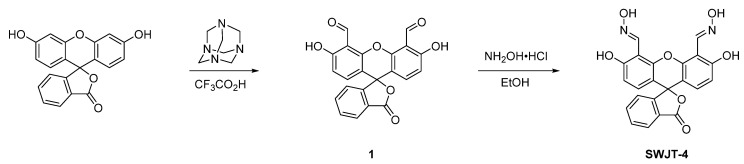
Synthesis of SWJT-4.

According to the Duff reaction, two aldehyde groups were formed at the *ortho* positions of two hydroxyl groups of fluorescein [42]. Two oxime groups as the recognition sites were then added to the fluorophore to construct **SWJT-4** (Scheme 1). The C = N rotation in **SWJT-4** would weaken the fluorescence. With the addition of DCP, an isoxazole or cyano group would be formed, which would inhibit the rotation of C = N and therefore enhance the fluorescence. The corresponding ^1^H and ^13^C NMR spectra and ESI-MS of **SWJT-4** are demonstrated in Figures S1–S3 (Supplementary Materials). In order to study its solvent effect on fluorescence properties of **SWJT-4**, five common organic solvents, namely acetone, methanol, tetrahydrofuran (THF), *N*,*N*-dimethyl formamide (DMF) and dimethyl sulfoxide (DMSO), were selected to test their performance under excitation at 520 nm. As shown in Figure S4 (Supplementary Materials), **SWJT-4** in DMSO has a longer emission wavelength. After the addition of DCP, the fluorescence intensity was enhanced clearly. Subsequently, the impact of pH on **SWJT-4** was also studied (Figure S5, Supplementary Materials). **SWJT-4** did not react with DCP under acidic conditions, and the best reaction condition was pH 7.0 to 8.0. Therefore, DMSO–HEPES (1/1, *v/v*, pH 7.4) buffer solution was determined as the optimal condition.

#### Fluorescence Response of **SWJT-4** to DCP

**Figure 1:**
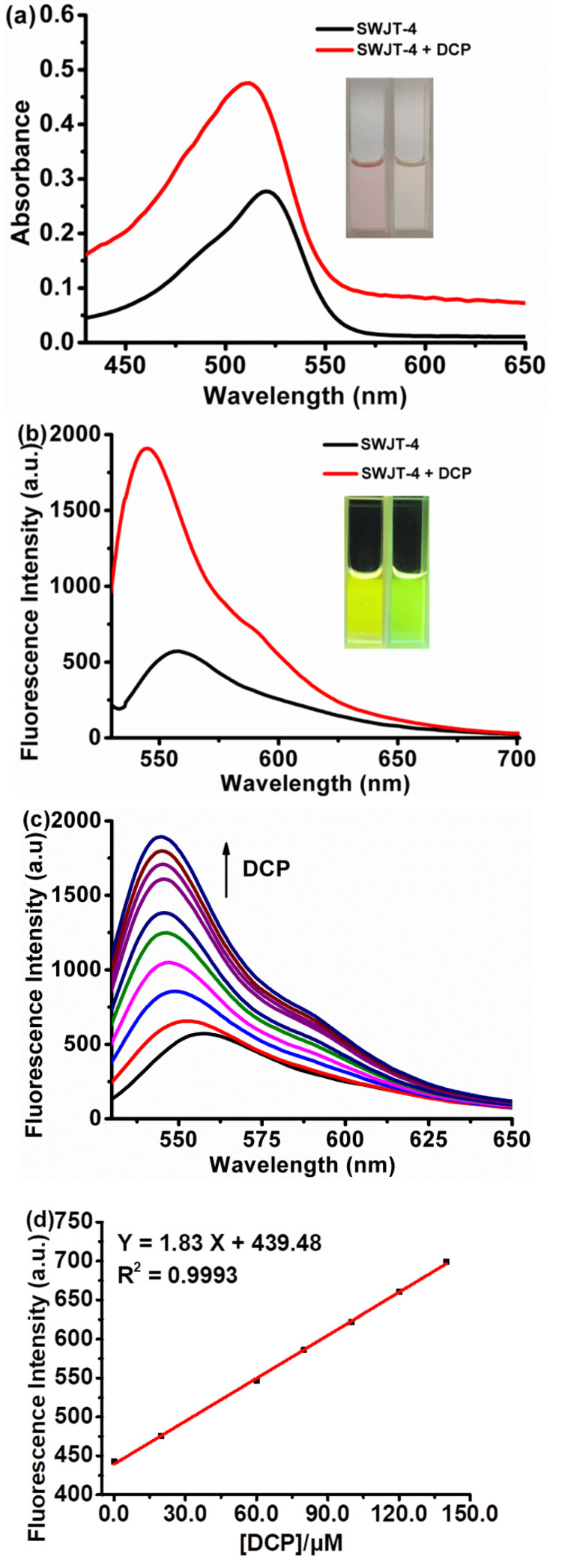
(**a**) The absorption spectrum of **SWJT-4** (10.0 µM) and **SWJT-4** + DCP (1.0 mM) in DMSO– HEPES (1/1, *v/v*, pH 7.4) buffer solution. Inset: the images of **SWJT-4** and **SWJT-4** after addition of DCP under visible light. (**b**) Fluorescence spectrum of **SWJT-4** (10.0 µM) and **SWJT-4** + DCP (1.0 mM) in DMSO–HEPES (1/1, *v/v*, pH 7.4) buffer solution. Inset: the images of **SWJT-4** and **SWJT-4** after addition of DCP under ultraviolet light. (**c**) Fluorescence titrations of **SWJT-4** (10.0 µM) with different concentrations of DCP (0–1000.0 µM); (**d**) Linear relationship between the fluorescence intensity of **SWJT-4** (10.0 µM) at 545 nm and the concentration of DCP (0–140.0 μM).

As shown in Figure 1a, the UV–visible absorption spectrum of **SWJT-4** exhibited prominent absorption at 522 nm, which was attributed to the formation of intramolecular hydrogen bonds between the hydroxyl groups on fluorescein and the nitrogen atom on oxime, and the hydrogen bonds would enlarge the conjugation of the probe [43]. When DCP was added to the solution of the probe, the absorbance was blue-shifted to 508 nm, which is the absorption of the ring-opening structure of fluorescein [44]. These results showed that the reaction occurred between **SWJT-4** and DCP to break the intramolecular hydrogen bonds. The color of the solution changed from pink to pale yellow (Figure 1a, inset). For the fluorescence spectrum, under the excitation of 520 nm, **SWJT-4** showed weak emission at 557 nm (*Φ* = 5.7%) (Figure 1b). After the addition of DCP, the fluorescence was enhanced at 545 nm (*Φ* = 26.7%) with a slight blue-shift [45]. The fluorescence color of the solution was observed to turn chartreuse to green (Figure 1b, inset). These results showed that **SWJT-4** was a turn-on fluorescent probe and could be used for the detection of DCP.

The fluorescence titration experiment of **SWJT-4** was then studied. As shown in Figure 1c, the fluorescence intensity gradually increased with the increase in DCP. There was a good linear relationship between DCP concentration and the fluorescence intensity in the range of 0–140.0 μM (Figure 1d). The detection limit was calculated as 53.0 nM (Figure S6, Supplementary Materials), which was much lower than the reported lethal dose (0.01 mg/L) [46]. This result indicated that **SWJT-4** had high sensitivity to DCP and could detect DCP at lower concentrations in an aqueous solution. Moreover, as shown in Figure S7 (Supplementary Materials), the recognition reaction finished within 100 min after the addition of DCP. The pseudo-first-order reaction constant (*k*_obs_) was 5.37 × 10^−4^ s^−1^. In order to verify the stoichiometry between the **SWJT-4** and DCP, a Job plot analysis was performed (Figure S8, Supplementary Materials), which indicated that the ratio of **SWJT-4** and DCP was 1:2. By Benesi–Hildebrand equation of this binding mode (1:2), a linear line with good linearity was obtained, and the binding constant was 1.37 × 10^6^ M^−2^ (Figure S9, Supplementary Materials). These results were in good agreement with the stoichiometry between chemodosimeter and DCP.

### 2.2. Competition Experiments

**Figure 2:**
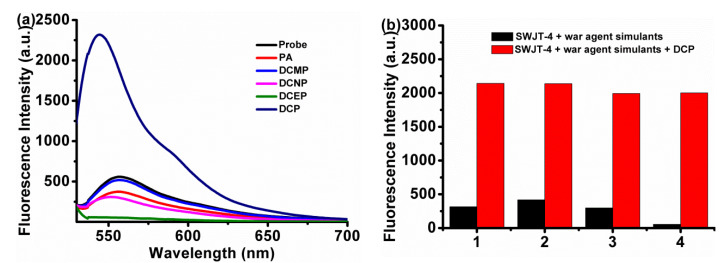
The fluorescence responses of (**a**) **SWJT-4** (10.0 μM) with different war agent simulants (1.0 mM). From left to right: **SWJT-4**, phosphoric acid (PA), cyanomethyl diethyl phosphate (DCMP), cyanoyl diethyl phosphate (DCNP), ethyl dichlorophosphate (DCEP) and diethyl chlorophosphate (DCP); (**b**) **SWJT-4** (10.0 μM) with different war agent simulants (1.0 mM) and DCP (1.0 mM) in DMSO–HEPES (1/1, *v/v*, pH 7.4) buffer solution (1, phosphoric acid; 2, cyanomethyl diethyl phosphate; 3, cyanoyl diethyl phosphate; 4, ethyl dichlorophosphate). Black bar: **SWJT-4** + war agent simulants; red bar: **SWJT-4** + war agent simulants + DCP.

In order to investigate the plausible interference of other organophosphorus reagents or nerve agent mimics on the detection of DCP, phosphoric acid (PA), cyanomethyl diethyl phosphate (DCMP), cyanoyl diethyl phosphate (DCNP) and ethyl dichlorophosphate (DCEP) were selected to study the selectivity of **SWJT-4** (Figure 2). With the addition of other phosphate-containing substances, the fluorescence intensity of **SWJT-4** changed little. However, after the addition of DCP to the solution of **SWJT-4**, the fluorescence at 545 nm was significantly enhanced. These results clearly showed that the fluorescent probe **SWJT-4** could recognize DCP effectively even in the presence of other war agent mimics.

### 2.3. Response Mechanism

**Figure 3:**
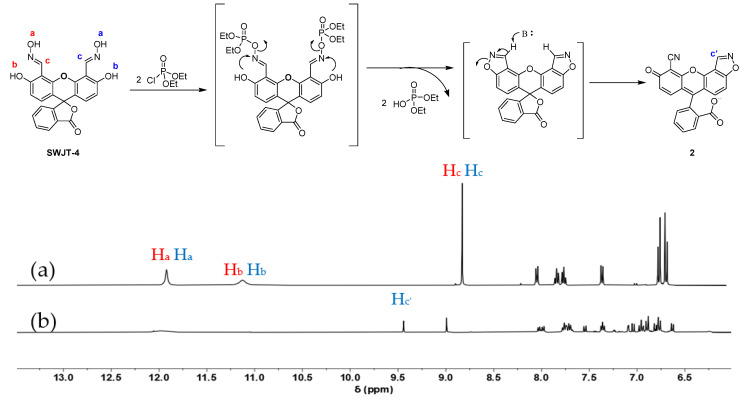
Partial ^1^H NMR spectra of (**a**) **SWJT-4** and (**b**) **SWJT-4** + DCP in DMSO-*d*_6_.

In order to identify the reaction mechanism between **SWJT-4** and DCP, ^1^H NMR titration was performed. As shown in Figure 3a, the peaks at 11.90 ppm and 11.10 ppm belonged to the proton signals of two hydroxyl groups (H_a_) on oxime moieties and two hydroxyl groups (H_b_) on fluorescein in **SWJT-4**, respectively. The chemical shift of the proton (H_c_) of the aldoxime group was at 8.80 ppm. After the addition of DCP, the original signals H_a_ and H_b_ in the probe disappeared, and one aldoxime proton H_c_ moved from 8.80 to 9.44 ppm downfield. The above results indicated that different reactions occurred at the two reaction sites of **SWJT-4** to form product **2**. One oxime group reacted with one DCP to form the nitrile [47], and the other oxime group reacted with another DCP to form an isoxazole ring. Firstly, the hydroxyl group in the oxime attacked the phosphorus center of DCP to form phosphate oxime. Then, another hydroxyl group in the adjacent position of the oxime group could intramolecularly attack this generated intermediate, and the isoxazole ring was then formed through a release of phosphate moiety [48]. Meanwhile, the mixture of **SWJT-4** and DCP was also characterized by ESI-MS, and the peak at *m*/*z* 381.3 corresponding to product **2** was observed (Figure S10, Supplementary Materials).

### 2.4. Computational Studies

**Figure 4:**
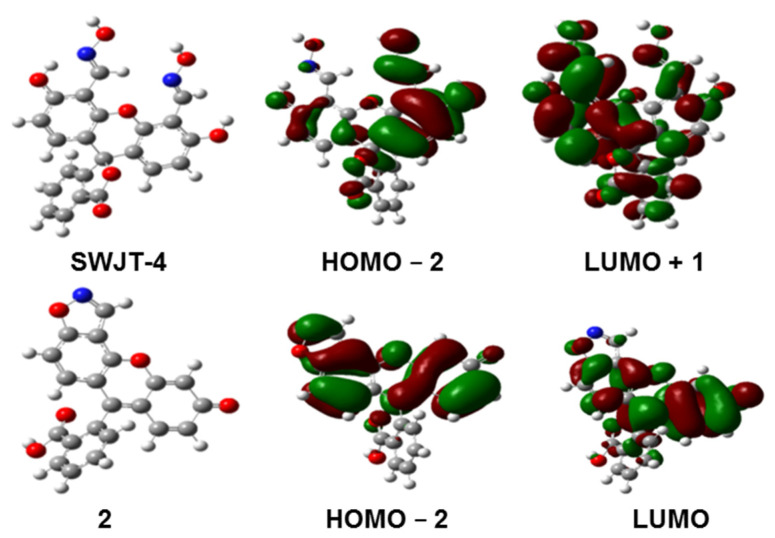
The optimized structures and the molecular orbital plots of **SWJT-4** and **2**.

In order to further study the fluorescence responses of **SWJT-4** with DCP and the influence of different solvent media on the absorption and emission, the B3LYP/6-31g method was conducted by Gaussian simulation (DFT) [49,50,51]. As shown in Figure 4, the main contribution for **SWJT-4** was from HOMO ‒ 2 to LUMO + 1. The electron clouds of their HOMO ‒ 2 orbital in **SWJT-4** were distributed on one benzene ring of the xanthrene group and the adjacent oxime group. The electron clouds of the LUMO orbital of **SWJT-4** were mainly distributed on the whole molecule instead. These results suggested the weak fluorescence of **SWJT-4**. As for product **2**, the main contribution was HOMO ‒ 2 to LUMO. The electron clouds of HOMO ‒ 2 and LUMO orbitals were all mainly distributed in the xanthrene moiety, indicating the strong fluorescence character of **2**. These results were very consistent with the fluorescence turn-on change on the detection of DCP using **SWJT-4**. Then, the absorption and emission of **SWJT-4** were calculated in DMSO or in water (Figure S11, Supplementary Materials). As shown in Figure S11a,c (Supplementary Materials), the maximum emission wavelength of **SWJT-4** in DMSO or in water was about 287 nm and the maximum absorption wavelength of **SWJT-4** in DMSO or in water was 280 nm (Figure S11b,d, Supplementary Materials). Although these wavelengths greatly differ from the measured results, the results indicated that the solvent effect had almost no effect on absorption and emission spectra in different solvent media.

### 2.5. Gas-Phase Detection of DCP

**Figure 5:**
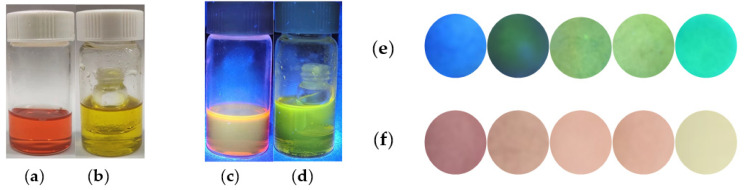
The color changes of (**a**,**c**) **SWJT-4** (10.0 M) (DMSO–HEPES, 1/1, *v/v*, pH 7.4) solution and (**b,d**) exposure to DCP vapors under visible light or ultraviolet light. Paper sensors that visually detect different concentrations of DCP under ultraviolet light (**e**) and visible light (**f**). From left to right: [DCP] = 0 mM, 1.0 × 10^−3^ mM, 1.0 × 10^−2^ mM, 1.0 × 10^−1^ mM, 1.0 mM.

Considering its practical application, DCP vapors were used to determine the recognition ability of **SWJT-4** (Figure 5a–d). The solution of **SWJT-4** was placed in a glass bottle with a lid (Figure 5a,c). The color of the solution was orange-pink under visible light (Figure 5a) and chartreuse in ultraviolet light (Figure 5c). As a contrast, the solution of **SWJT-4** was placed in another glass bottle with a lid, in which there was a smaller bottle containing DCP (Figure 5b,d). When the DCP vapors came into contact with the solution of **SWJT-4**, the color of the solution changed from orange-pink to yellow in visible light (Figure 5b), and the chartreuse color turned to green under ultraviolet light (Figure 5d). These results indicated that **SWJT-4** could detect DCP vapor.

Moreover, the responses of **SWJT-4** loaded on filter paper to different concentrations of DCP vapor were studied. With the increase in DCP concentration, the color of filter paper changed obviously under ultraviolet (Figure 5e) or visible light (Figure 5f), which was observed by the naked eye. The green color in paper sensors gradually increased under ultraviolet light (Figure 5e), and under visible light, the color of the paper sensors changed from pink to pale-yellow (Figure 5f). These results showed that the probe can be used as a fluorescent test paper to detect DCP vapor and has potential application in the development of detection kits for DCP.

## 3. Materials and Methods

### 3.1. Materials and Reagents

Related materials, reagents and the detail of detection are described in the Supplementary Materials.

### 3.2. Synthesis of Probe **SWJT-4**

The compound **1** was synthesized according to a known procedure [42]. Hydroxylamine hydrochloride (24.4 mg, 0.62 mmol) was dissolved in ethanol (3 mL) and stirred at room temperature for 10 min. Then compound **1** (60.4 mg, 0.16 mmol) was dispersed in 5 mL of ethanol and dripped into the above solution. The reaction mixture was stirred at room temperature for 2 h. The organic solvent was removed by rotary evaporation, and the crude product was isolated by column chromatography (dichloromethane:methanol = 80:1) on silica gel to obtain the probe **SWJT-4** (54.2 mg, yield 82.9 %) as a pink solid. ^1^H NMR (400 MHz, DMSO-*d*_6_): *δ* = 11.90 (s, 2H), 11.10 (s, 2H), 8.80 (s, 2H), 8.02 (d, *J* = 7.6 Hz, 1H), 7.81 (t, *J* = 7.5 Hz, 1H), 7.74 (t, *J* = 7.0 Hz, 1H), 7.34 (d, *J* = 7.5 Hz, 1H), 6.74 (d, *J* = 8.8 Hz, 2H), 6.67 (d, *J* = 8.8 Hz, 2H). ^13^C NMR (100 MHz, DMSO-*d*_6_): *δ* = 169.0, 159.1, 152.5, 149.0, 145.6, 136.3, 130.8, 130.1, 126.4, 125.3, 124.5, 113.6, 110.2, 106.1, 82.6 ppm. ESI-MS: *m*/*z* 419.1 [M + H] ^+^.

## 4. Conclusions

In summary, the fluorescence changes of a novel fluorescent probe **SWJT-4** for the detection of DCP based on the dual reaction site were explored. **SWJT-4** showed good selectivity for DCP and the obvious color change in an aqueous solution. Notably, the two reaction sites in the probe also triggered different reaction types. Moreover, **SWJT-4** could be used for DCP vapor detection and as fluorescent test paper to detect DCP.

## Data Availability

Not applicable.

## Supplementary Materials

The following supporting information can be downloaded online: Table S1: Some reported work for the detection of DCP. Figures S1–S3: Copies of ^1^H,^13^C NMR, ESI-MS spectra of **SWJT-4**. Figure S4: The changes of fluorescence in different solvents. Figure S5: The effect of pH on fluorescence intensity. Figure S6: The linear relationship between **SWJT-4** and different concentrations of DCP. Figure S7: Time-dependent experiment of **SWJT-4**. Figures S8 and S9: Job’s and Benesi-Hildebrand plots of **SWJT-4** with DCP. Figure S10: ESI-MS spectrum of **SWJT-4** + DCP. Figure S11: The fluorescence spectrum and absorption spectrum of **SWJT-4** calculated by DFT in DMSO and water.
