# Supplementary material for: A Double-Site Chemodosimeter for Selective Fluorescence Detection of a Nerve Agent Mimic
Source: Molecules. 2022 Jan 13;27(2):489. doi: 10.3390/molecules27020489 (PMC8780492; doi:10.3390/molecules27020489)
Supplement: Supplementary file 1 [file molecules-27-00489-s001.zip › molecules-1538349-supplementary.pdf]

A Double-Site Chemodosimeter for Selective Fluorescence Detection of a Nerve Agent Mimic

Xin Guo [1], Chang-Xiang Liu [1], Yuan Lu [2], Ya-Wen Wang [1,*] and Yu Peng [1,*]

[1] School of Life Science and Engineering, Southwest Jiaotong University, Chengdu 610031, China

[2] Chengdu Municipal Bureau of Economic and Information Technology

* Correspondence: ywwang@ swjtu.edu.cn; pengyu@ swjtu.edu.cn

Table of Contents

1. General Methods......S2
2. Some reported work for the detection of DCP (**Table S1**)......S3
3. ^{1}H, ^{13}C NMR spectra and ESI-MS of **SWJT-4** (**Figures S1-S3**)......S5
4. Fluorescence intensity of **SWJT-4** in different solvents (**Figure S4**)......S7
5. The effect of pH on fluorescence intensity (**Figure S5**)......S8
6. The linear relationship between **SWJT-4** and different concentrations of DCP (**Figure S6**)......S9
7. Time-dependent experiment of **SWJT-4** (**Figure S7**)......S10
8. Job's and Benesi-Hildebrand plots of SWJT-4 with DCP in DMSO/HEPES buffer solution (**Figures S8** and **S9**)......S11
9. ESI-MS spectrum of **SWJT-4** + DCP (**Figure S10**)......S12
10. The fluorescence spectrum and absorption spectrum of **SWJT-4** calculated from DFT in DMSO and water (**Figure S11**)......S13

1. General Methods.

All the reagents such as Fluorescein, diethyl chlorophosphate, diethyl cyanophosphonate, hexamethylenetetramine (HMTA), trifluoroacetic acid (TFA) and hydroxylamine hydrochloride were from Beijing Innochem Co., Ltd,. All solvents were purchased by the analysis of the printed chemicals of Tianjin Tianzheng Fine Chemical Reagent Factory. For fluorescence spectroscopy, DMSO is the spectral grade.

^{1}H and ^{13}C NMR spectra were measured by Bruker Avb-400 spectrometer, TMS was used as internal reference, and ESI-MS spectra were measured on Bruker ESQuire 6000 spectrometer. The fluorescence spectra were recorded by Hitachi Pharmaspecc's F4600 spectral fluorometer.

Various stock solutions (1.0 mM) of the phosphoric acid (PA), cyanomethyl diethyl phosphate (DCMP), cyanoyl diethyl phosphate (DCNP), ethyl dichlorophosphate (DCEP) and diethyl chlorophosphate (DCP) in DMSO were prepared. The Stock of SWJT-4 (1.0 mM) was prepared in dimethyl sulfoxide. Test solutions were prepared by placing 20.0 μL of the probe stock solution into a test tube, and then diluting to 2.0 mL with DMSO/HEPES buffer (1/1,v/v, pH 7.4)solution, followed by the addition of an appropriate aliquot of war agent simulants stock solution. For all measurements, fluorescence spectra were obtained by excitation at 520 nm. Fluorescence quantum yields were determined in solution, using fluorescein (Φ = 0.85 in 0.1 M NaOH) as a standard.

2. Some reported work for the detection of DCP.

Table S1. A comparison about ours and some reported work for the detection of DCP.

Probe		Solvent	Ex (nm)	LOD (nM)	Reference
**A**	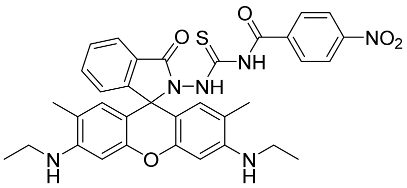	DMF (5% Et_3N)	538 nm	142.0 nM	1
**B**	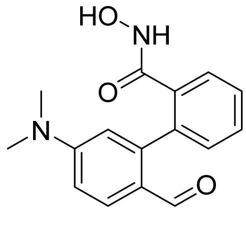	Acetonitrile	365 nm	10.4 nM	2
**C**	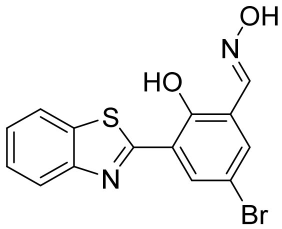	DMF	385 nm	33.5 nM	3
**D**	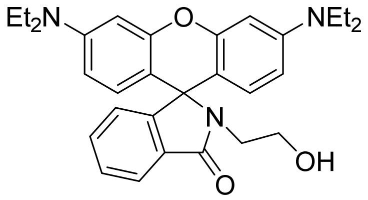	DMF	520 nm	–	4
**E**	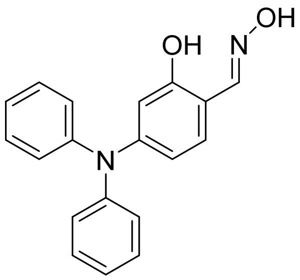	CH_3CN-HEPES (4:6, v/v, pH 7.4)	379 nm	140 nM	5
**F**	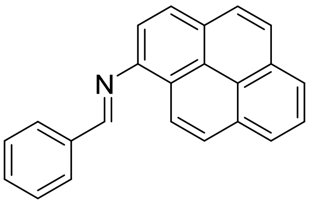	H_2O-CH_3CN (5:95, v/v)	382 nm	–	6

G	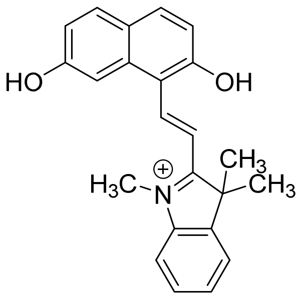	CH_3CN-HEPES (1 : 1, v/v, pH 7.4)	354 nm	29.5 nM	7
**SWJT-4**	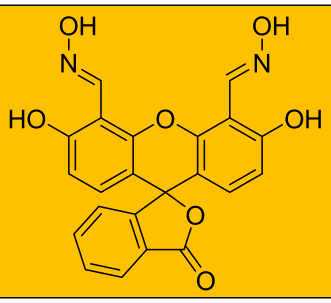	DMSO-HEPES (1/1, v/v, pH 7.4)	520 nm	**53.0 nM**	**This work**

1. So, H. S.; Angupillai, S.; Son, Y. A., Prompt liquid-phase visual detection and low-cost vapor-phase detection of DCP, a chemical warfare agent mimic. *Sens Actuators B Chem.* **2016**, 235, 447-456.
2. Huo, B.; Du, M.; Shen, A.; Li, M.; Lai, Y.; Bai, X.; Gong, A.; Yang, Y., "Covalent-Assembly"-Based Fluorescent Probe for Detection of a Nerve-Agent Mimic (DCP) via Lossen Rearrangement. *Anal Chem.* **2019,** 91, 10979-10983.
3. Guria, U. N.; Maiti, K.; Ali, S. S.; Gangopadhyay, A.; Samanta, S. K.; Roy, K.; Mandal, D.; Mahapatra, A. K., An Organic Nanofibrous Polymeric Composite for Ratiometric Detection of Diethyl Chlorophosphate (DCP) in Solution and Vapor. *ChemistrySelect.* **2020,** 5, 3770-3777.
4. Wu, W. H.; Dong, J. J.; Wang, X.; Li, J.; Sui, S. H.; Chen, G. Y.; Liu, J. W.; Zhang, M., Fluorogenic and chromogenic probe for rapid detection of a nerve agent simulant DCP. *Analyst* **2012,** 137, 3224-3226.
5. Ali, S. S.; Gangopadhyay, A.; Pramanik, A. K.; Guria, U. N.; Samanta, S. K.; Mahapatra, A. K., Ratiometric sensing of nerve agent mimic DCP through in situ benzisoxazole formation. *Dyes Pigm.* **2019,** 170, 107585.
6. Kim, H. J.; Jang, S.; Ren, W. X.; Bartsch, R. A.; Sohn, H.; Kim, J. S., Imine-functionalized, turn-on fluorophore for DCP. *Sens. Actuators, B.***2011,** 153, 266-270.
7. Ali, S.S.; Gangopadhyay, Maiti, K.; Mondal, S.; Pramanik, A. K.; Guria, U. N.; Uddin, M. R.; Mandal, S.; Mandal, D.; Mahapatra, A. K., A chromogenic and ratiometric fluorogenic probe for rapid detection of a nerve agent simulant DCP based on a hybrid hydroxynaphthalene–hemicyanine dye. *Org. Biomol. Chem.* **2017,** 15, 5959-5967.

3. ^{1}H, ^{13}C NMR spectra and ESI-MS of SWJT-4.

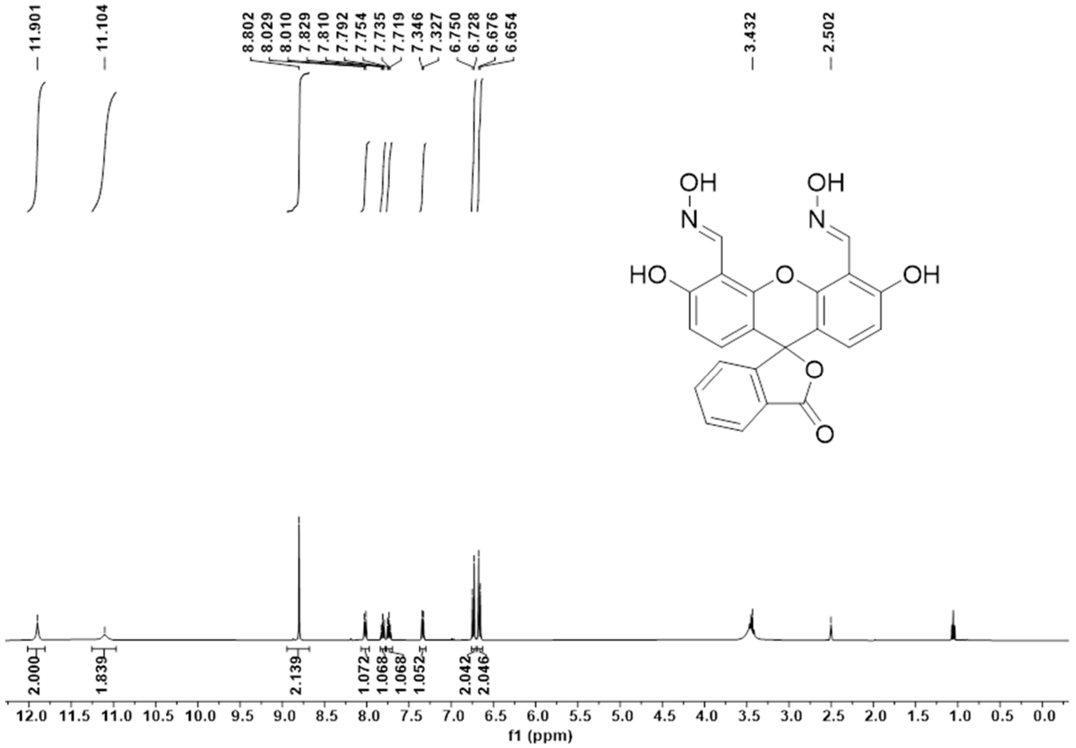

Figure S1. ^{1}H NMR spectrum (400 MHz, DMSO-d_6) of **SWJT-4**.

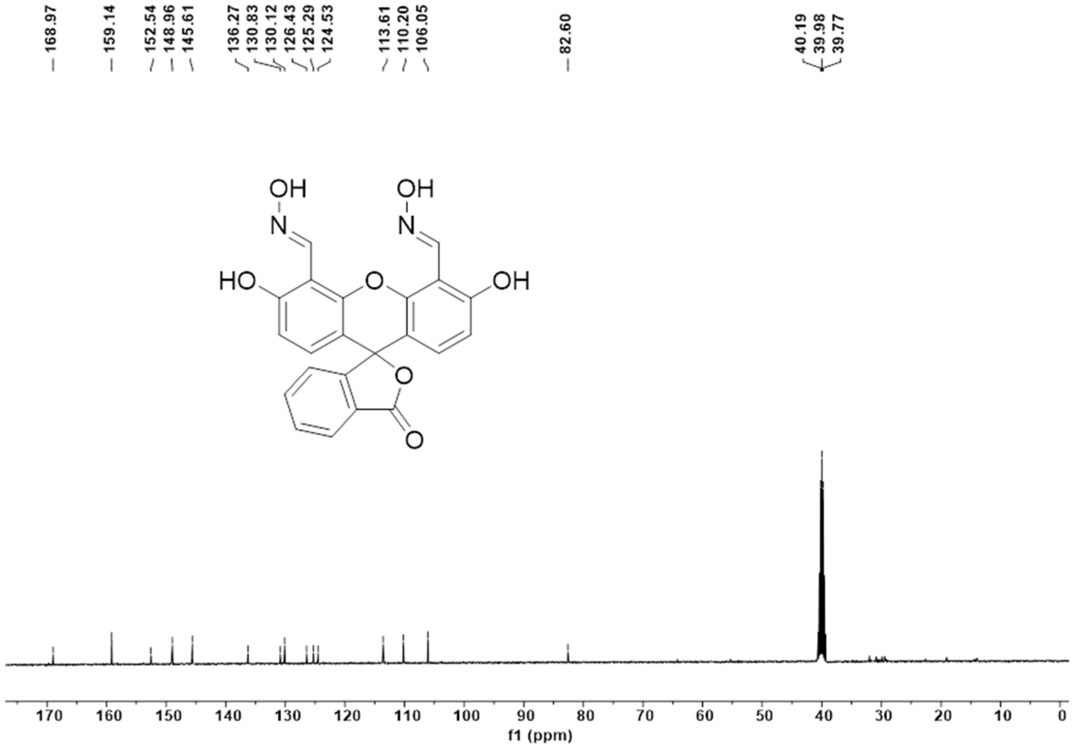

Figure S2. ^{13}C NMR spectrum (100 MHz, DMSO-d_6) of **SWJT-4**.

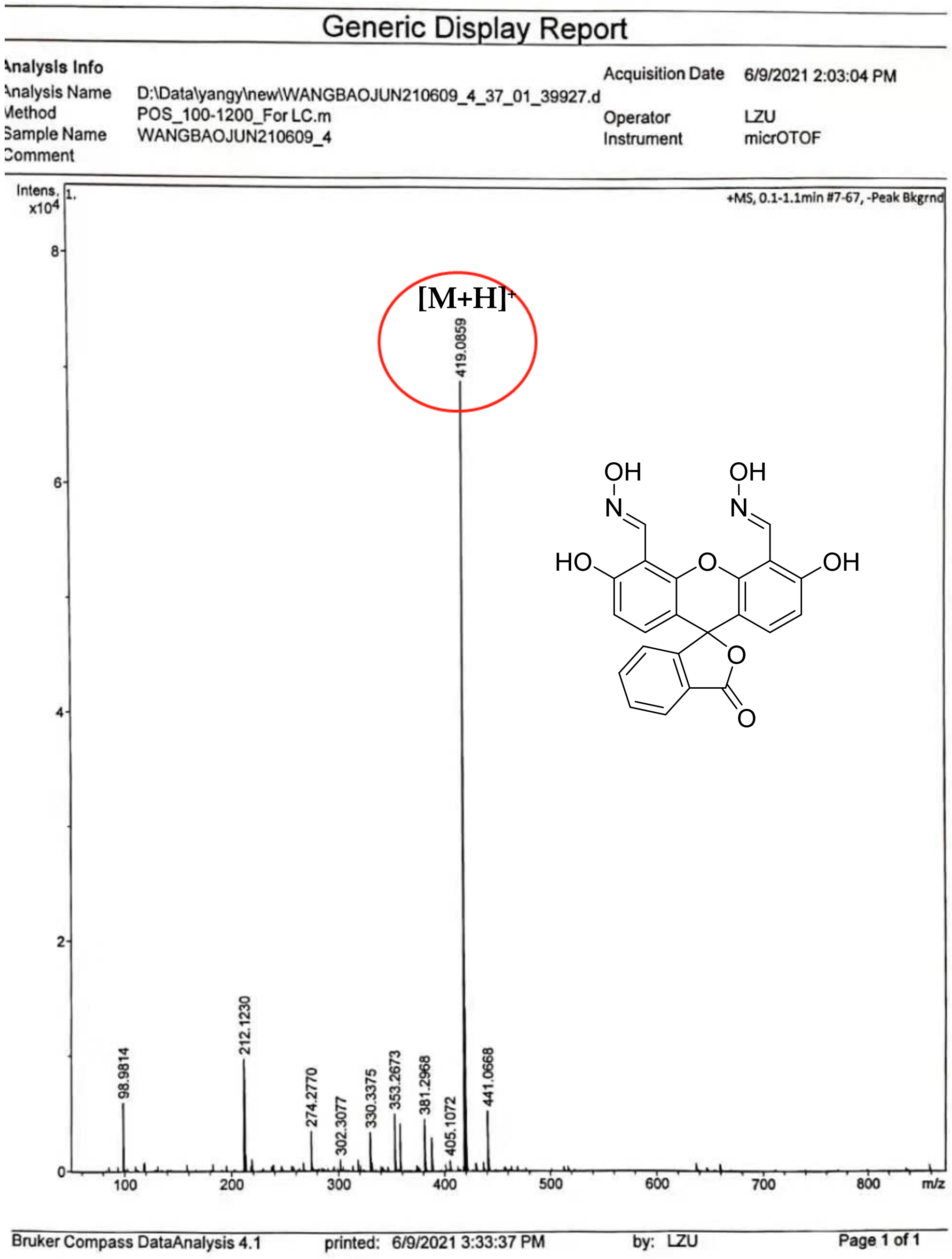

Figure S3. ESI-MS spectrum of **SWJT-4**.

4. Fluorescence intensity of SWJT-4 in different solvents.

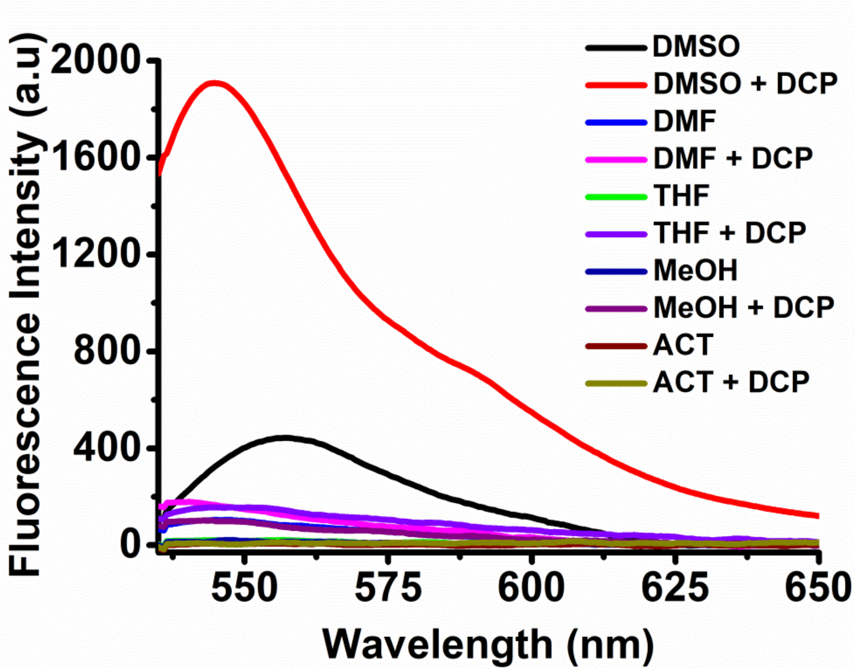

Figure S4. The fluorescence emission spectra of **SWJT-4**(10.0 μM) and **SWJT-4** + DCP (1.0 mM) in acetone (ACT), methanol (MeOH), tetrahydrofuran (THF), N, N-dimethylformamide (DMF) and dimethyl sulfoxide (DMSO).

5. The effect of pH on fluorescence intensity.

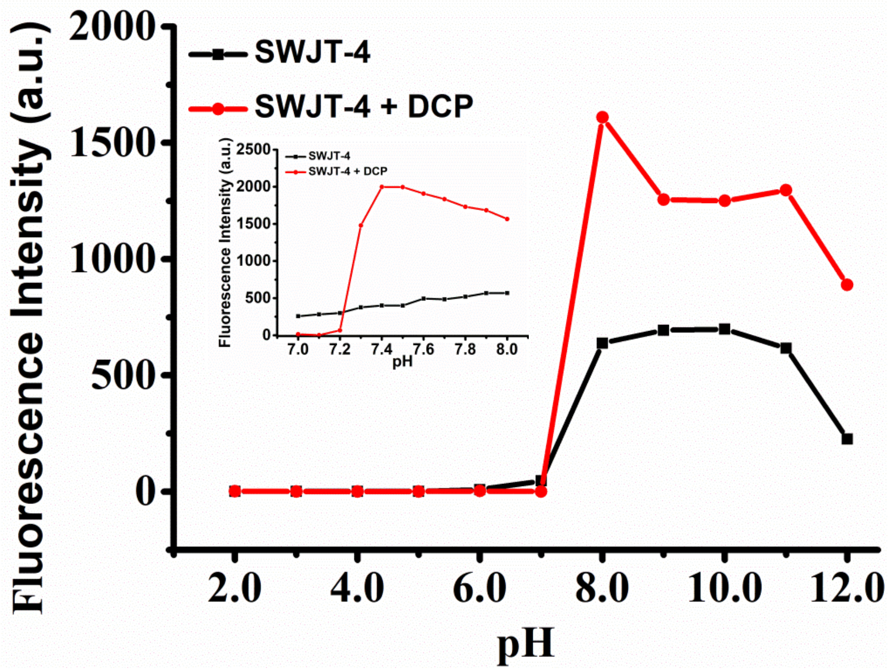

Figure S5. The fluorescence intensity of **SWJT-4** (10.0 μM) at 545 nm in the presence and absence of DCP (1.0 mM) in DMSO/HEPES buffer (1/1, v/v) under different pH (2.0 - 12.0).

6. The linear relationship between SWJT-4 and different concentrations of DCP.

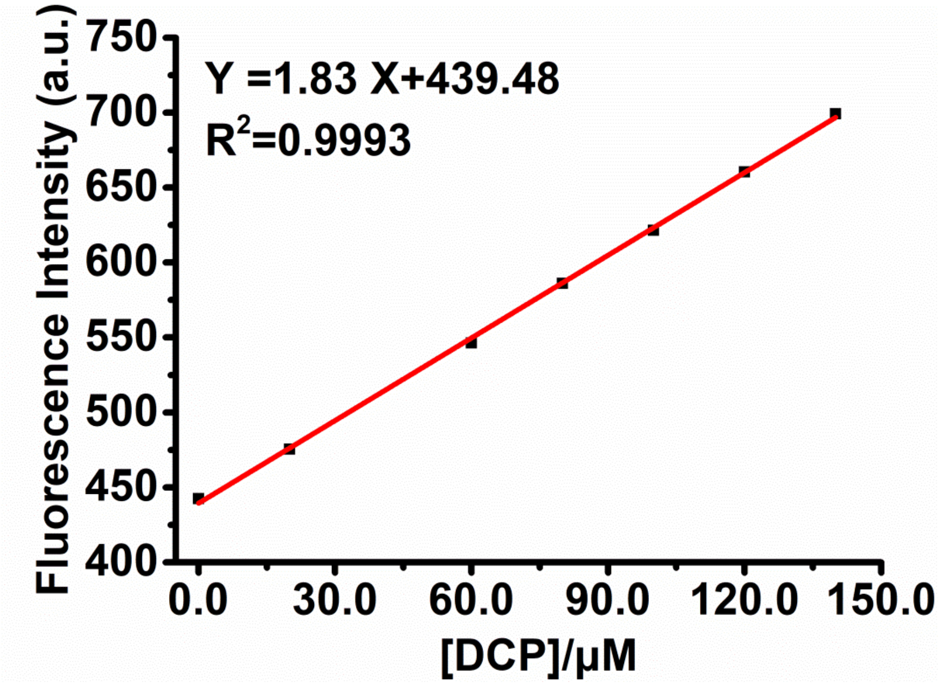

Figure S6. Linear relationship between the fluorescence intensity of **SWJT-4** at 545 nm and the concentration of DCP (0-140.0 μM).

The limit of detection (LOD) was calculated from the following equation based on fluorescence titration results:

LOD = K ×δ/S

Where K = 2 or 3 (2 was taken in this case), δ is the standard deviation of the blank solution, and *S* is the slope of the straight-fit line.

Linear Equation: Y=1.83X + 439.48

S = 439.48 δ= 0.032 K = 3

LOD = K ×δ/S = 53 nM

7. Time-dependent experiment of SWJT-4.

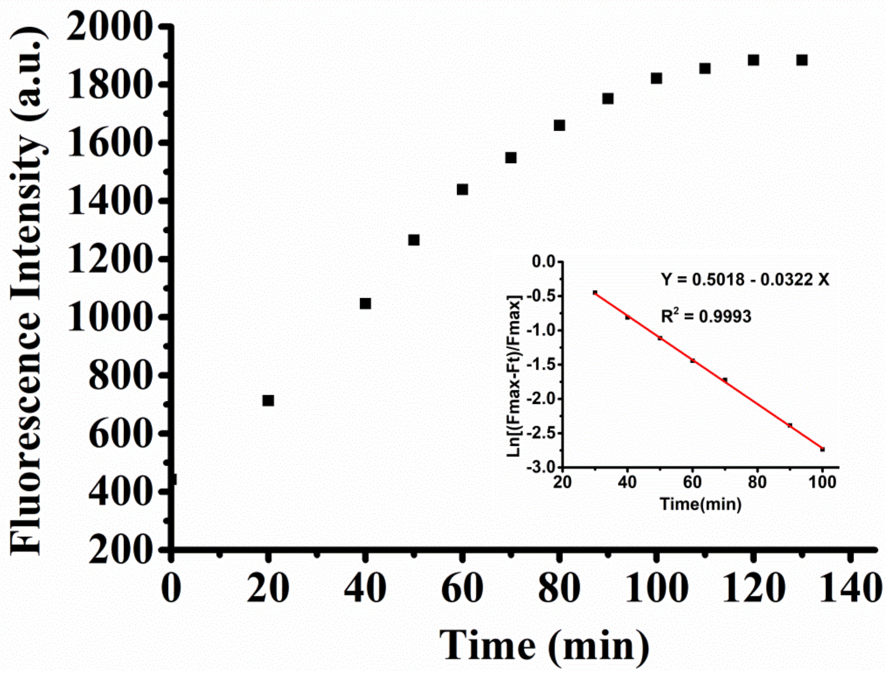

Figure S7. Time dependence of fluorescence spectrum of **SWJT-4** (10.0 μM) in DMSO : HEPES = 1 : 1 (v/v) solution at pH 7.4 (λ_{ex} = 520 nm). Inset: The pseudo-first-order kinetics equation of **SWJT-4**.

The corresponding kinetic constants were determined using the following equations:

$\ln [(F_{max} - F_t)/F_{max}] = - k_{obs}t$

$t_{1/2} = \ln2/k_{obs}$

Where F_{max} and F_t are the fluorescence intensity at maximum emission and time t.

$k_{obs} = 5.37 \times 10^{-4}\ s^{-1}$

8. Job's and Benesi-Hildebrand plots of SWJT-4 with DCP in DMSO/HEPES buffer solution.

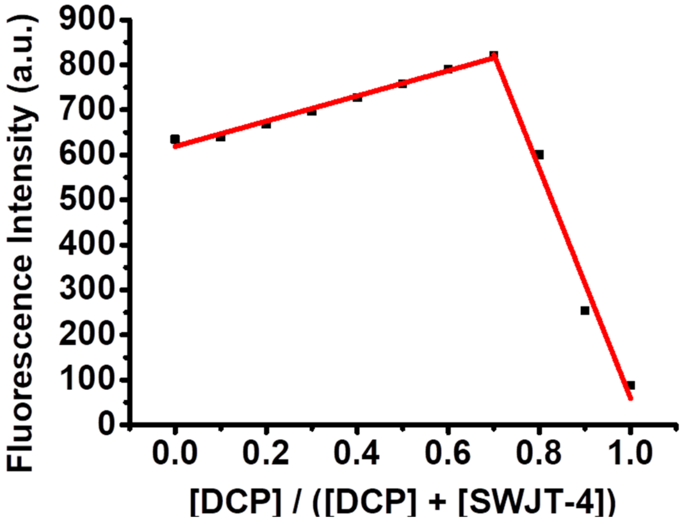

Figure S8. Job's plot for **SWJT-**4 with DCP in DMSO-HEPES (1/1, v/v, pH 7.4) buffer solution.

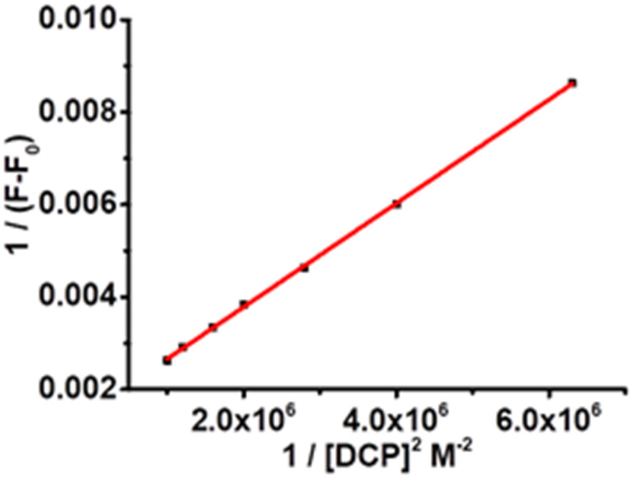

Figure S9. Benesi-Hildebrand plot of 1, assuming 1:2 stoichiometry for association between **SWJT-4** and DCP in DMSO-HEPES (1/1, v/v, pH 7.4) buffer solution.

The binding constant was determined using a reported procedure for a 1:2 binding mode. The result of the analysis as follows:

Equation: $Y = A + B \times X$

$Y = 0.00154 + 1.12214 \times 10^{-9} \times X$

$R = 0.9997$

$K = A / B = 1.37 \times 10^{6}\ M^{-2}$

9. ESI-MS spectrum of SWJT-4 + DCP.

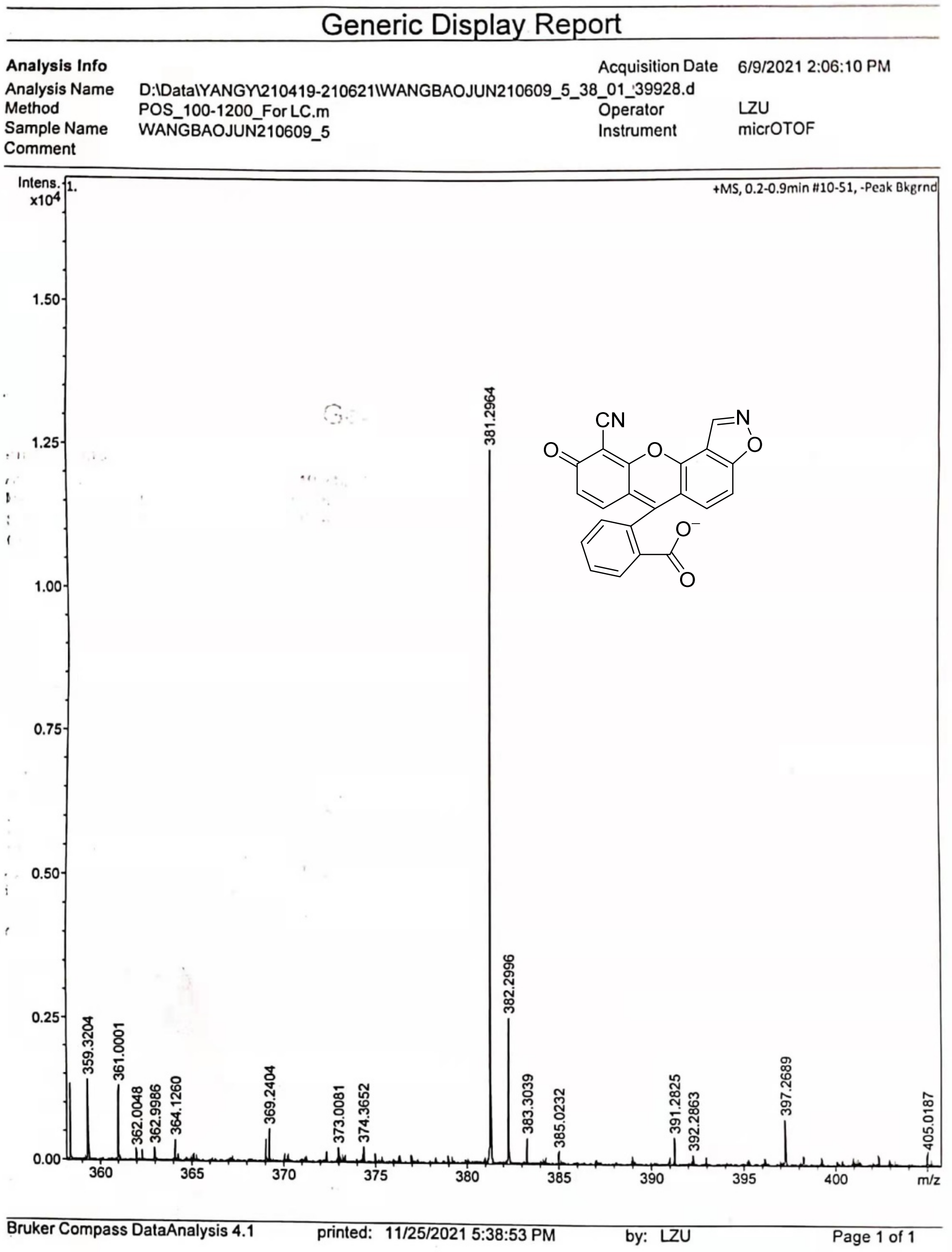

Figure S10. ESI-MS spectrum of **SWJT-4** + DCP.

10. The fluorescence spectrum and absorption spectrum of SWJT-4 calculated from DFT in DMSO and water.

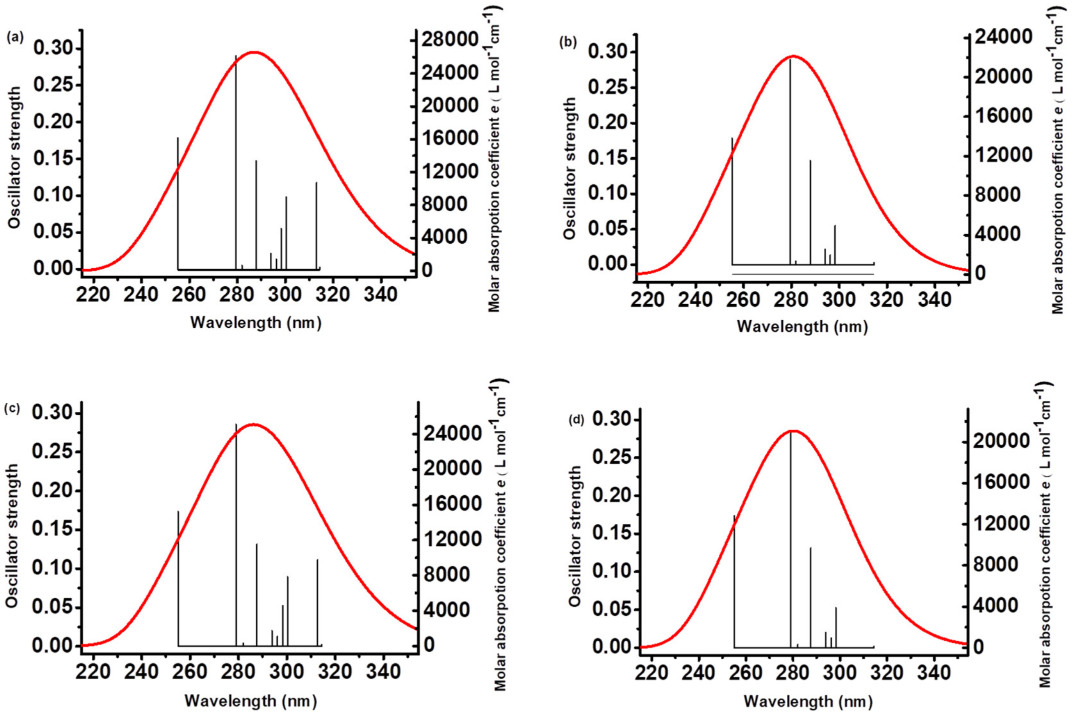

Figure S11. The fluorescence spectrum (a) and absorption spectrum (b) of SWJT-4 calculated from DFT in DMSO; (c) The fluorescence spectrum and absorption spectrum (d) of SWJT-4 calculated from DFT in water.
